# An Evaluation of the Relationship Between the Mesiobuccal Canal Configuration, the Interorifice Distance, and the Root Lengths of the Permanent Maxillary First Molars with Cone Beam Computed Tomography

**DOI:** 10.3390/diagnostics14232703

**Published:** 2024-11-30

**Authors:** Mehmet Ozgur Ozemre, Hazal Karslıoglu, Kıvanc Kamburoglu

**Affiliations:** 1 Department of Dentomaxillofacial Radiology, Faculty of Dentistry, Mersin University, Mersin 33343, Turkey; 2 Department of Dentomaxillofacial Radiology, Faculty of Dentistry, Baskent University, Ankara 06110, Turkey; hazal23k64@gmail.com; 3 Department of Dentomaxillofacial Radiology, Faculty of Dentistry, Ankara University, Ankara 06560, Turkey; dtkivo@yahoo.com; 4 Faculty of Stomatology, Department of Surgery and Pediatric Dentistry, Akhmet Yassewi International Kazakh Turkish University, Turkestan 161200, Kazakhstan

**Keywords:** anatomy, canal configurations, endodontics, mesiobuccal canal, maxillary first molar, cone beam computed tomography

## Abstract

Background/Objectives: This study aimed to investigate the relationship between the mesiobuccal root canal configuration (MB RCC), the interorifice distance (IOD) and the corresponding root and other root lengths of the permanent maxillary first molars; Methods: Cone beam computed tomography (CBCT) images were acquired between 2020 and 2023 for different purposes unrelated to this study. Overall, 1550 CBCT images were retrospectively evaluated. A dentomaxillofacial radiologist with 15 years of experience evaluated the CBCT images and performed the measurements; Results: According to the MB RCC, there was no statistically significant difference between the Vertucci type II and Vertucci type IV groups in terms of the mean age and sex distribution (*p* = 0.694 and *p* = 0.273). There was no statistically significant difference in the IOD between the MB RCC groups (*p* = 0.755). Moreover, according to the MB RCC, there was no statistically significant difference between the Vertucci type II and Vertucci type IV groups in terms of the mesiobuccal, distobuccal, palatinal, and mean root lengths (*p* > 0.05); Conclusions: There was no association between the IOD and the type of RCC in the maxillary first molars. New studies conducted by collecting data from different centers to explore the different morphological features of maxillary first molars and detect their anatomical differences will provide more reliable and accurate results.

## 1. Introduction

**Figure 1:**
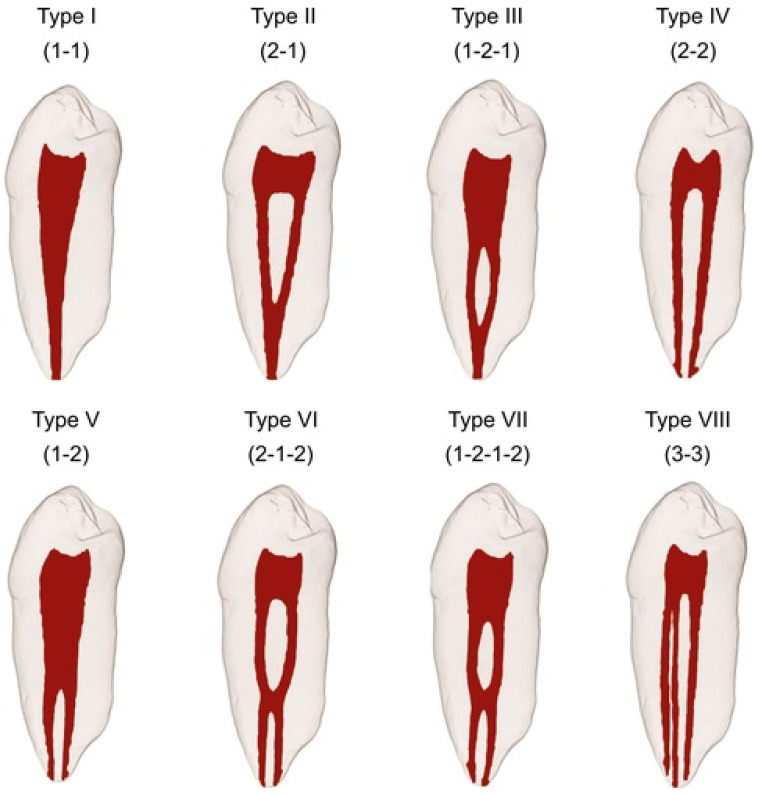
Diagrammatic representations of Vertucci’s classification of the root canal morphology [3].

The root canal structure is highly complex and diverse. There are many studies in the literature that classify the root canal morphology in different ways. Vertucci studied the root canals of human teeth in great detail and developed a standardized method to classify the root canal variations into eight different types [1]. This classification system is widely used in many studies (Figure 1).

Adequate knowledge of the root and root canal morphology and their possible variations is essential for excellent endodontic treatment [2]. The lack of consideration of the root canal structure may cause an endodontic treatment to fail [3]. Given the large individual, genetic, and ethnic diversity, additional canals should be sought [4]. Several studies in different ethnic populations (American, Iranian, Turkish, Chinese, and Jordanian) have shown that there are differences in the root canal morphology of the permanent teeth [5,6,7,8,9,10]. In a study by Amos [11] on a series of full-mouth radiographs from 1000 patients, it was found that some mandibular first premolars had double canal systems and that this was more common in African American patients than in Caucasian patients. However, patients from both of these groups were not included in this study. Trope et al. [12] found significant differences in the number of roots and canals between African American and White patients. The results of both studies showed that African American patients tended to have a larger number of roots and canals compared to Caucasian patients.

The success of endodontic treatment is ensured via thorough chemical and mechanical preparation, thorough filling of the endodontic space, proper cavity preparation for shaping and cleaning procedures, and proper hermetic restoration to surround the root canal in three dimensions and prevent microleakage. The permanent maxillary molars have an especially complex root and canal anatomy. Therefore, most studies on the root canal morphology have focused on the mesiobuccal (MB) roots of the permanent maxillary first molars [2,13,14,15,16,17]. A review of the literature shows that the presence of a second mesiobuccal (MB2) canal in the maxillary first molars is reported in 50–90% of cases [15,18]. In studies using cone beam computed tomography (CBCT), the incidence of MB2 was 85.4% [16]. These canals may merge (Vertucci type II) or may have independent exit portals (Vertucci type IV). Therefore, the detection and localization of the MB2 canal is critical for the success of endodontic treatment.

In the literature, many different methods have been used to evaluate the root and canal morphology. Laboratory techniques include demineralization [19], the injection of Indian ink [20], hematoxylin staining [21], the use of Chinese ink [22], metal casting [23], in vitro radiography [24], macroscopic examination [25], grinding or sectioning [26,27], and scanning electron microscopy (SEM) [28]. Operating microscopy and conventional radiography are used in many stages of endodontic treatment. Computed tomography (CT), micro CT, spiral CT, and CBCT have been used in clinical trials. However, variations in biological and methodological factors have resulted in differences in the reported prevalence rates. Moreover, it has been reported that using a surgical microscope during the treatment of the maxillary molars does not significantly increase the number of second mesiobuccal canals found, as is the case when access is altered and no microscope is used. Microcomputed tomography, staining, and translucency techniques can detect most MB2 canals in the maxillary molars; however, such studies have been performed on extracted human teeth [29]. Periapical radiography is the most commonly used imaging method for the study of the dental anatomy and endodontic diagnosis. However, this provides only limited 2D information that is sensitive to the superposition of the anatomical structures and the distortion of the images due to the angulation used; therefore, it is not a reliable method for the detection of accessory canals [30]. CBCT, specifically designed for dentistry, has a higher image resolution as well as a much lower radiation dose compared to CT. This technique has proven useful in evaluating the root canal morphology and detecting periapical lesions in the maxillary region. CBCT provides sufficient information about the root and canal morphology in different directions, with no anatomical superposition, which cannot be achieved when using 2D X-rays or clinically [29,31]. Table 1 shows a comparison of the advantages and disadvantages of the most used radiographic methods. Sousa et al. [32] reported that the diagnostic accuracy of CBCT was 0.89, while that of periapical radiographs was 0.55. The corresponding values for specificity were 0.93 and 0.98, while those for sensitivity were 0.18 and 0.79. Despite the advantages of CBCT, it also has some limitations that can affect the image quality and the detection of MB2. These limitations include patient movement during exposure, the presence of restorations in the same dental arch, and a high bone density. The major problems affecting the diagnostic accuracy and interpretation of CBCT images are beam hardening artifacts and the scattering created by radiopaque materials such as restorations, retrograde fillings, metal pin-posts and root fillings, and high-density adjacent structures such as bone or enamel [33].

The clinician’s detailed knowledge of the mesial buccal root length of the maxillary first molars before starting endodontic treatment enables them to predict the root canal configuration that may be encountered during mechanical–chemical preparation and reduces the likelihood of iatrogenic errors. Most studies have shown that the distance between the root canal orifices determines the RCC [16,34,35,36,37]. In the literature, only one study has investigated the relationship between the root length and RCC using CBCT images [35].

This study aimed to evaluate the relationship between the MB RCC, the IOD, and the corresponding root and other root lengths of the permanent maxillary first molars. If there is a relationship between them, it will increase the success of endodontic treatment, especially in the permanent maxillary molars.

## 2. Materials and Methods

### 2.1. Sample and Assessment of CBCT Images

The CBCT images used in this study were selected from the archives of the Department of Dentomaxillofacial Radiology, Faculty of Dentistry, Başkent University. This study was approved by the Baskent University Ethics Committee (D-KA2207). Since it was a retrospective archive review, informed consent was not required. The study was conducted under the principles of the Declaration of Helsinki.

CBCT images were acquired between 2020 and 2023 for different purposes unrelated to this study, such as implant planning, endodontic evaluation, orthodontic treatment, and the surgical removal of affected teeth. In this study, 1550 CBCT images were retrospectively evaluated. The inclusion criteria consisted of CBCT scans of participants of either sex between the ages of 18 and 51 years, in which the MB2 canal was clearly visualized in the permanent maxillary first molars. The exclusion criteria included teeth with periapical lesions, resorption, canal calcification, large coronal restorations, cemented posts, or prior root canal treatment. A total of 1106 first molars met these criteria: 508 maxillary right first molars and 598 maxillary left first molars. In accordance with the statistical recommendations, 106 teeth, including 24 (2%) maxillary first molars of other Vertucci types, such as III, V, VI, and VII, were excluded from the study. Finally, 500 maxillary right first molars and 500 maxillary left first molars were included.

All CBCT images were acquired using the Morita 3D Accuitomo 170 model from Japan. The following parameters were used to acquire the images: 5 mA; 90 kVp; and voxel sizes of 0.08, 0.125, and 0.25 mm. The field of view (FOV) dimensions were 40 × 40, 60 × 60, 80 × 80, and 100 × 50 mm. The i-Dixel software (version 2.2.1.6), developed by Morita, was used for the analysis process, and the analysis was performed on a medical monitor, the Radiforce MX270W model, manufactured by the Eizo Corporation.

Two dentomaxillofacial radiologists with 7–15 years of experience evaluated the inclusion and exclusion criteria together and performed calibration for the evaluation of the MB2 canal in the CBCT images. They classified 1106 first molars together according to their Vertucci types. After this, a more experienced dentomaxillofacial radiologist performed the measurements. The same images of 100 teeth were reevaluated by the same examiner after 30 days to calculate the intraobserver agreement, which was found to be high (ICC: 0.91). To evaluate the interobserver agreement, 20% of all CBCT images were analyzed by the other dentomaxillofacial radiologist. The interobserver agreement was also high (ICC: 0.90)

#### 2.1.1. Measurements of Root Length

**Figure 2:**
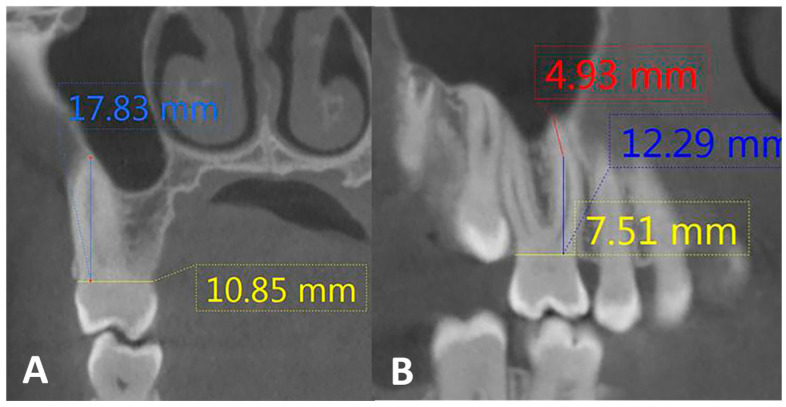
CBCT reconstructions of MB root in maxillary first molar tooth. Root length measurement in (**A**) coronal section, (**B**) curved MB root.

The images were analyzed in the coronal and sagittal planes. A line connecting the buccal and palatal cementoenamel junctions (CEJs) was used as the reference plane. The point at which this line met the uppermost point of the root was determined as the root length (Figure 2A). Measurements were taken in both sections, and their averages were considered. Curved roots were measured according to Moidu et al. [10]. Measurements were obtained from the CEJ to the first point of curvature, followed by the length of the tangent from the curvature to the highest point on the root (Figure 2B). Both measurements were performed to determine the final root length.

#### 2.1.2. Measurements of IOD

**Figure 3:**
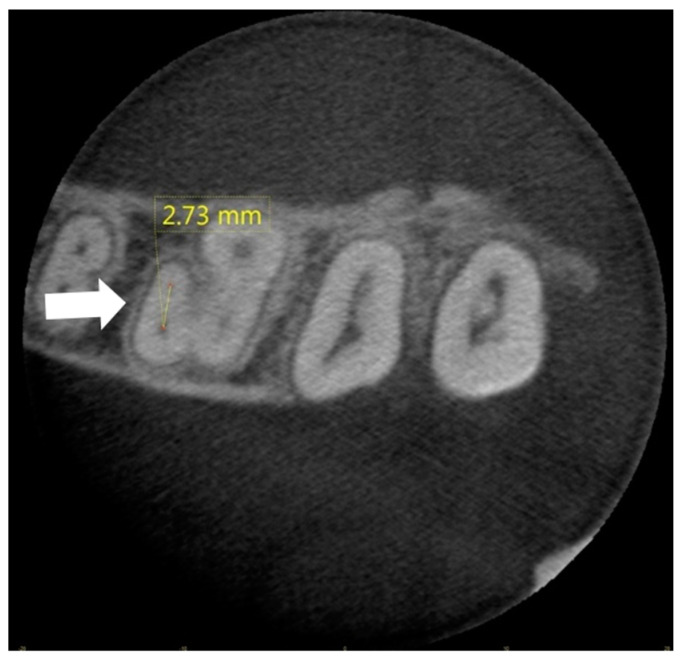
CBCT reconstructions of MB root in maxillary first molar tooth. First axial section in which MB2 canal was visualized. IOD was measured between MB1 and MB2 canals.

For the IOD measurement, the axial section in which the MB2 canal was first displayed was considered. The line joining the centers of MB1 and MB2 was considered as the IOD (mm) (Figure 3).

#### 2.1.3. Assessment of RCC

**Figure 4:**
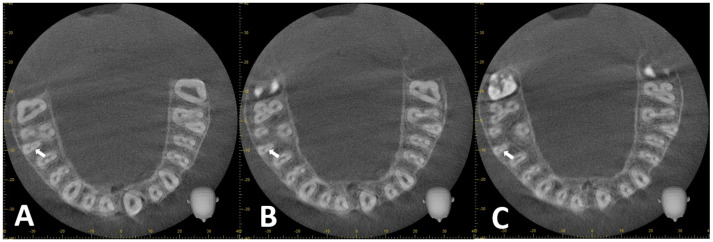
CBCT reconstructions of MB root (arrow) in maxillary first molar tooth. (**A**–**C**) Coronal to apical axial sections exhibiting Vertucci type IV RCC.

**Figure 5:**
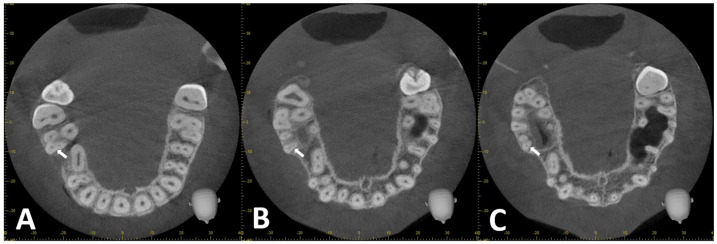
CBCT reconstructions of MB root in maxillary first molar tooth. (**A**–**C**) Coronal to apical axial sections exhibiting Vertucci type II RCC.

To determine the RCC, the axial section was evaluated from the CEJ to the root apex. A Vertucci type IV RCC was considered if two radiolucent canal lines were visualized along the root length (Figure 4A–C). Vertucci type II RCC was considered if the outlines of the two radiolucent canals converged (Figure 5A–C).

### 2.2. Statistical Analysis

The IBM SPSS Statistics (version 25.0) software, developed by the IBM Corporation in the USA, was used to analyze the data. The Kolmogorov–Smirnov test was used to determine whether the continuous numerical data conformed to a normal distribution, and the homogeneity of variance was examined via Levene’s test. Descriptive statistics are presented as the mean ± standard deviation or the median (25th and 75th percentiles) for continuous numerical variables, and the number of cases and percentages (%) for categorical variables. The mean differences between groups were evaluated using Student’s *t*-test, and the differences between continuous numerical data were evaluated using the Mann–Whitney U test when the parametric test assumptions were not met. Pearson’s χ2 test was used to evaluate categorical data. Spearman’s correlation test was used to determine the relationships between the continuous numerical variables. The level of statistical significance was determined as *p* < 0.05.

## 3. Results

The descriptive statistics for the clinical and demographic characteristics of the patients included in this study are presented in Table 2. A total of 1000 maxillary first molars (478 females (47.8%) and 522 males (52.2%)) were analyzed from 1550 CBCT images. Of these, it was determined that 652 had a Vertucci type II (65.2%) and 348 had a Vertucci type IV (34.8%) canal configuration in the mesiobuccal canal morphology. The interorifice distance was found to be 2.41. The mesiobuccal and distobuccal root lengths were the same. As expected, the palatal root length was greater than the root length. These values are listed in Table 2. The average length of all three roots was found to be 12.44.

When we evaluated the mesiobuccal canal morphology, the interorifice distance in Vertucci type II cases was 2.41. It was measured as 2.39 in Vertucci type IV cases. The mesiobuccal root length was determined as 12.17 in patients with a Vertucci type II morphology and 12.22 in patients with a Vertucci type IV morphology. The distobuccal root length was found to be the same in those with Vertucci type II and type IV morphologies. In patients with a type II morphology, the palatal root length was 12.90. It was measured as 12.89 in patients with a type IV morphology. The average root length in patients with a Vertucci type II morphology was measured as 12.43 and was shorter than that in patients with a Vertucci type IV morphology.

One thousand teeth were evaluated regarding the clinical and demographic characteristics of the cases with a mesiobuccal RCC (Table 3). There was no statistically significant difference between the type II and type IV groups in terms of the mean age or sex distribution (*p* = 0.694 and *p* = 0.273, respectively). Furthermore, no statistically significant difference in the IOD was found between the mesiobuccal RCC groups (*p* = 0.755).

Regarding the mesiobuccal RCC, no statistically significant difference between the Vertucci type II and Vertucci type IV groups was found for the mesiobuccal, distobuccal, palatal, and mean root lengths (*p* > 0.05).

Table 4 shows the significance levels of the mesiobuccal, distobuccal, palatinal, and mean root lengths in relation to the IOD. Finally, no statistically significant correlation was found between the IOD and the mesiobuccal, distobuccal, palatal, or mean root length (*p* > 0.05).

## 4. Discussion

The endodontic treatment of the maxillary first molars is considered challenging due to the complex root and canal anatomy, as well as the high prevalence of the MB2 canal [38]. It is hypothesized that the main reason for the poor long-term prognosis following root canal treatment in the permanent maxillary molars is the failure to detect the MB2 canal. In the literature, the endodontic treatment failure rates for an MB2 canal that was not filled with canal sealant range from 46% to 78%. This emphasizes the importance of the detection and treatment of the MB2 canal [33].

In endodontics, the most commonly used methods to detect the second root canal are visual evaluation, 2D X-ray, and dental operating microscopy. In clinical practice, periapical radiography is the most commonly used method to evaluate the root canal morphology. This method provides additional information with a low radiation dose and cost. However, despite its frequent use for endodontic diagnosis and treatment, periapical radiography cannot fully demonstrate the complex anatomical structures of the teeth due to the overlapping inherent in two-dimensional images. The use of CBCT in endodontic diagnosis and treatment planning makes it possible to evaluate the root canal anatomy in 3D before endodontic treatment [32]. The American Association of Endodontists and the American Academy of Oral and Maxillofacial Radiology have published guidelines justifying the use of CBCT, especially for teeth with a complex root canal anatomy [33]. In addition, the European Society of Endodontology recommends the use of CBCT with a limited field of view for teeth with a complex root canal anatomy that require retreatment. To increase the visibility of the root canal anatomy, a reduced voxel size and increased scan time in high-resolution mode are preferred. A smaller field of view increases the CBCT image resolution and reduces the radiation exposure required among patients [39,40]. Bauman et al. [41] reported that voxel sizes of ≤0.2 mm are optimal for the better detection of MB canals. The present study did not classify the CBCT images according to the FOV areas. This can be considered one of the most important limitations of our study. All measurements were performed on the coronal and sagittal sections of the CBCT images. Compared to the gold standard, CBCT-based length measurements have been shown to be more reliable and accurate.

Numerous authors have compared CBCT imaging with other methods used to evaluate the root canal morphology. Matherne et al. [42] compared the effectiveness of CBCT imaging with photostimulated phosphor plate radiography and a charge-coupled device and found that KIBT was better than other methods in morphologic identification. Domark et al. [43] found no significant difference between micro-CT imaging and CBCT imaging for canal detection in permanent maxillary molars. Blattner et al. [44] compared the CBCT results with dental cross-sectional results and reported that there was no difference in their accuracy.

When the results of recent studies on the prevalence of MB2 canals are compared with the results of previous CBCT studies, it is found that the rate of MB2 canals is much higher in recent studies. The observation of a much larger proportion of MB2 canals may be due to advances in CBCT technology. It may also depend on the level of experience of the clinician evaluating the volumes or the interpretation of the CBCT volumes. There are many studies in the literature showing that clinicians interpret radiographs in different ways [45,46]. In a study by Parker et al., examining the ability to diagnose periapical lesions on CBCT images in various groups of individuals and comparing this with the interpretations of dentomaxillofacial radiologists, it was reported that the level of clinical experience was associated with the ability to correctly diagnose the disease [46].

Ethnic and genetic differences are thought to be the causes of the differences in the rates and configurations of the MB2 canal in the maxillary first molars. However, the age range of the patient groups is also an important factor. Kiefner et al. [47] reported that secondary dentin deposition in elderly patients can significantly narrow the root canal space, leading to canal calcification. Reis et al. [48] showed that the prevalence of the MB2 canal in the maxillary molars decreases with age and the canal moves towards the apical region; this is attributed to increased dentin deposition on the root canal walls with age. Martins et al. [17] found a higher prevalence of MB2 canals in males, which is in agreement with previous studies showing differences between the sexes. In the literature, no studies show a higher prevalence of MB2 canals in women. Martins et al. [17] found a higher prevalence of MB2 in younger patients, confirming previous findings. The lower rates of MB2 in older patients are thought to be due to the fact that preexisting MB2 root canals are not visible on CBCT due to closure or narrowing.

Clinicians should possess sufficient knowledge of the RCC to overcome these challenges. Understanding the relationship between the root length and root canal morphology may aid clinicians in treating root canals. In the literature, only one study focused on this topic, and it concluded that an increased root length is associated with a Vertucci type 2 RCC [35]. Moreover, no study has assessed the relationship between the RCC and the IOD, MB, DB, or palatal root length of the permanent maxillary first molars.

The rationale behind this study lay in the results of a previous CBCT study in which a statistically significant association was found between the root length and root canal configuration. The results of the study conducted by Mouid et al. [35] showed that shorter roots exhibited the presence of two separate canals (type IV RCC). A type II RCC was observed as the length of the roots increased. While Moudi et al. evaluated 100 patients, we evaluated 1000 patients; in addition, we measured the lengths of three roots. Since we observed in our study that the tooth root length did not affect the RCC, we believe that it offers a valuable contribution to the literature on this subject. In addition, it has been documented that the distance between the root canal orifices determines the RCC. Cimilli et al. [34] found, in the mesial roots of the mandibular first molars, that the interorifice distance (IOD) was significantly greater for a Vertucci type IV compared with a type II RCC. This fact has not been confirmed regarding the MB roots of the maxillary first molars. Both Moidu’s work and our investigations suggest that the interorifice distance in the maxillary first molars does not determine the root canal configuration.

Moidu et al. [35] found that the length of the MB root is a very important parameter in the prediction of RCC types. Vertucci type II RCCs have been observed in roots with increased lengths [35]. In contrast, in the present study, there was no statistically significant difference between the type II and type IV groups in terms of the mesiobuccal, distobuccal, palatal, and mean root lengths (*p* > 0.05). The RCC is racially and genetically determined; therefore, its incidence varies among ethnic populations [35,49,50]. This may be one of the reasons that the results obtained in this study differ from those of Moidu et al. [35]. Another reason may be the different sample sizes of the studies; the number of images included in the study by Moidu et al. was smaller than that in the current study.

In addition, Habib and Howait [33] found that the mean distance between the MB and MB2 canals at the pulpal floor was 2.52 ± 0.76 mm. This finding provides insights into the location of the MB2 orifice relative to the MB orifice on the pulp floor in the Saudi subpopulation. When the results of a study by Su et al. [51] were analyzed, it was found that there was an average interorifice distance of 1.91 ± 0.59 mm in the Taiwanese population, while Moidu et al. [35] reported a range of 2.58 ± 0.04 mm in the Indian population.

Previously, it was shown that, in the mesial roots of the mandibular first molars, the IOD was significantly greater for a Vertucci type IV than for a type II RCC [34]. However, Moidu et al. [35] found no association between the IOD and the RCC type in the mesiobuccal roots of the maxillary first molars. Similarly, there was no significant difference between the type II and type IV groups in terms of the IOD in the present study. The canals tend to remain separate throughout their entire length only when the IOD is >3 mm [21]. In our study, the mean IOD was <3 mm. This may explain why no relationship between the IOD and the RCC types in the maxillary first molars was found.

Our study had some limitations. This was a retrospective study with a limited sample size that was representative of the Turkish population. The root canal morphology may differ among individuals living in different geographical regions and having different ethnic backgrounds. This is extremely important when comparing the MB2 rates in different CBCT prevalence studies. Therefore, when the patient demographics vary widely, comparisons between studies should be performed with caution. This challenge can be overcome by ensuring that the same observer evaluates the CBCT databases from different countries, keeping the age constant, and applying a 1:1 sex ratio. Furthermore, the root canal morphology is dynamic and can change over time. It may be difficult to determine the ideal patient age to best represent each region. Moreover, this study did not aim to examine the ethnicities of the patients. Therefore, additional studies including different populations and with larger sample sizes are needed. Another possible problem is the difference in CBCT interpretation between the observers. Despite efforts to calibrate the observers’ assessment skills by sharing images, references, and training videos and ensuring identical timelines, each observer’s past experience may also be influential.

To the best of our knowledge, this is the first study to evaluate the relationship between the RCC and the IOD, MB, DB, and palatal root lengths in the permanent maxillary first molars. In this study, we aimed to provide a preoperative prediction of the RCC based on the root length. However, there were no statistically significant differences in terms of the mesiobuccal, distobuccal, palatal, or mean root lengths between the type II and type IV groups. In addition, there were no statistically significant differences between the mesiobuccal RCC groups regarding the IOD.

In conclusion, there is no association between the IOD and the type of RCC in the maxillary first molars. In addition, the length of the roots is not an important anatomic parameter for the prediction of the type of RCC. New studies conducted by collecting data from different centers to explore the different morphological features of maxillary first molars and detect their anatomical differences will provide more reliable and accurate results. In addition, using a CBCT device with a limited field of view and a smaller voxel size improves the image resolution and image quality, allowing a more detailed examination of the root and canal anatomy. Further studies with different ethnicities and larger sample sizes are warranted.

## Figures and Tables

**Table 1:** A comparison of the advantages and disadvantages of the most used radiographic methods.

	Advantages	Disadvantages
Periapical [30]	Better resolution than panoramic	Only limited 2D information
OPG [29,30,31,32,33]	Radiation dose is lower than in CBCT	Only limited 2D information, superposition
CBCT [29,31,32,33]	3D information	Beam hardening artifacts and scattering

**Table 2:** Clinical and demographic characteristics of patients included in study.

	*n* = 1000
Age (years)	31.4 ± 7.6
Age range (years)	18–51
Sex	
Female	478 (47.8%)
Male	522 (52.2%)
Tooth number	
16	500 (50.0%)
26	500 (50.0%)
Mesiobuccal canal morphology	
Tip 2	652 (65.2%)
Tip 4	348 (34.8%)
Interorifice distance	2.41 (2.14–2.71)
Mesiobuccal root length	12.20 (11.93–12.38)
Distobuccal root length	12.20 (11.93–12.36)
Palatinal root length	12.89 (12.38–13.27)
Mean root length	12.44 (12.15–12.60)

**Table 3:** Demographic and clinical characteristics of cases according to mesiobuccal canal morphology.

	Tip 2 (*n* = 652)	Tip 4 (*n* = 348)	*p*-Value
Age (years)	31.3 ± 7.5	31.6 ± 7.7	0.694
Sex			0.273
Female	300 (46.0%)	178 (51.1%)	
Male	352 (54.0%)	170 (48.9%)	
Tooth number			
16	318 (48.8%)	182 (52.3%)	
26	334 (51.2%)	166 (47.7%)	
Interorifice distance	2.41 (2.24–2.71)	2.39 (1.96–2.72)	0.755
Mesiobuccal root length	12.17 (11.90–12.36)	12.22 (11.93–12.40)	0.605
Distobuccal root length	12.20 (11.93–12.36)	12.20 (11.93–12.36)	0.458
Palatinal root length	12.90 (12.39–13.28)	12.89 (12.37–13.25)	0.651
Mean root length	12.43 (12.15–12.62)	12.45 (12.17–12.59)	0.938

*p* < 0.05 was statistically significant.

**Table 4:** Correlation coefficients and significance levels between interorifice distance and root lengths.

	*n*	Correlation Coefficient	*p*-Value
Mesiobuccal	1000	−0.071	0.115
Distobuccal	1000	0.008	0.855
Palatinal	1000	0.062	0.166
Average	1000	−0.023	0.602

*p* < 0.05 was statistically significant.

## Data Availability

The raw data supporting the conclusions of this article will be made available by the authors on request.
